# Quantitative measurement of morphometric indicators of skeletal muscle cell behaviour and quality

**DOI:** 10.1098/rsif.2024.0634

**Published:** 2025-04-16

**Authors:** David Hardman, Katharina Hennig, Inês Belo Martins, William Roman, Edgar R. Gomes, Miguel O. Bernabeu

**Affiliations:** ^1^ Centre for Medical Informatics, The University of Edinburgh Usher Institute of Population Health Sciences and Informatics, Edinburgh, UK; ^2^ Universidade de Lisboa Instituto de Medicina Molecular João Lobo Antunes, Lisbon, Portugal; ^3^ Gulbenkian Institute for Molecular Medicine, Lisbon, Portugal; ^4^ Australian Regenerative Medicine Institute, Clayton, Victoria, Australia; ^5^ Victoria Node, European Molecular Biology Laboratory, Clayton, Victoria, Australia

**Keywords:** muscle cell, quantification, acetylcholine receptor, myonuclei, myotube

## Abstract

*In vitro* culturing of effective human-induced pluripotent stem cell-derived skeletal muscle cells (hiPSC-SMCs) has proven to be challenging. Progress is hindered by the limited range of metrics applied to assess experimental success. We present a semi-automated workflow for segmenting, tracking and quantifying migration and fusion behaviour in live and static images of myoblast and myotube cells. Workflow outputs are validated against manually labelled images and the metrics applied to images from case studies of *in vitro* cultures of primary mouse muscle cells under varying culture media conditions, mouse primary cells undergoing optogenetic stimulation and hiPSC-SMC. We show culture media-dependent differences in cell fusion dynamics and increased acetylcholine receptors in myonuclei under optogenetic stimulation. We show that myoblasts have greater persistence and proliferation in primary mouse cells than hiPSC, and cell–cell fusion occurred earlier but at a steadier rate in primary mouse cells.

## 1. Introduction

Culturing skeletal muscle tissue from human-induced pluripotent stem cell-derived skeletal muscle cells (hiPSC-SMCs) promises innovations in animal-free drug trials and precision medicine-guided therapeutics [1]. However, replicating the physiological structures of mature muscle cells cultured from precursor myoblast cells is currently a major obstacle; indeed, *in vitro* culturing of skeletal muscle tissue lags behind other tissue cultures, especially from human-derived cells [2].

*In vitro* muscle differentiation involves the cultivation of myoblasts, which are muscle progenitor cells capable of proliferating and differentiating into mature muscle fibres. Under growth-promoting conditions, myoblasts proliferate, but when switched to differentiation-inducing conditions—typically by reducing serum concentration and providing extracellular matrix support—they exit the cell cycle and begin expressing muscle-specific genes, such as *MyoD* and *myogenin*. During this differentiation process, myoblasts elongate, align with one another and undergo membrane fusion to form multi-nucleated myotubes, mimicking the structure of mature muscle fibres. The resulting myotubes further mature by organizing contractile proteins into sarcomeres, the functional units of muscle contraction, providing a valuable model to study muscle physiology and pathologies in a controlled environment.

Quantitative cell image analysis can provide information concerning the maturity, health and behaviour of cells [3]. Of specific interest when defining protocols for culturing muscle cells is maximizing myogenic potency [4,5]. Defining and quantifying key metrics of cell morphology and behaviour associated with myogenic potency during differentiation would allow for early assessment of experimental success. A further application is to elucidate the effects of interventions such as drug trials, co-culturing muscle cells with other cell types or characterizing the morphology of cells with specific muscular disorders [6]. Analysis of the large sample sizes of cells required to make confident conclusions about cell morphologies or behaviours, as well as the large amounts of imaging data provided via high-throughput cell screening, means that manual methods for quantification of muscle cells are not practical and that automated, or semi-automated, methodologies are required. Currently, image analysis of skeletal muscle cells tends to be qualitative or rely on the calculation of the fusion index (FI), the ratio of myonuclei to total muscle cell nuclei at a given time point, alone. FI represents only one aspect of myogenic potency [4] and can be misleading when not taken in context of further metrics of cell densities over time [7].

Existing cell-tracking software, such as Trackmate for FIJI software [8], is limited by the nature of myoblast differentiation, which requires a distinction between mononucleated myoblasts and the multi-nucleated syncytium of myotubes. Recent studies [4,7,9] have sought to distinguish between precursor cells and myotubes and introduce further quantitative metrics of myogenic potency. In these studies, cell nuclei with a DNA-binding staining such as DAPI or Hoechst are defined as belonging to a myotube if they lie within regions stained for proteins associated with muscle structure, such as myosin heavy chain or α-actinin. This method is sufficient for use on isolated myotubes, but for *in vitro* studies with high densities of myotubes and precursor myoblasts that coincide in the same space, the overlap of cell types results in false labelling of cells. This leads to inaccuracies in metrics without careful manual checking of the data. Even when labelling of nuclei is validated, this method gives only a binary indicator of differentiation and does not define, for example, cells which are differentiated but not yet fused, or cells which have recently fused. Also, to the authors’ knowledge, there are currently no image analysis workflows which quantify all key aspects of early-stage myogenesis, namely muscle precursor cell dynamics, muscle cell fusion potential, myotube morphology and myotube protein patterning.

We present a toolkit, using ImageJ and MATLAB software, for obtaining a suite of metrics of key early-stage cell quality indicators and cell behaviours for quantifying skeletal muscle cell differentiation *in vitro*. For live bright-field images, an in-house cell tracking system is applied to differentiate between myotube and precursor myoblast cells and obtain metrics of entropy and directionality in myoblast motion. In static images, myotubes and myoblasts are distinguished by overlaying cell nuclei stained with a DNA-binding fluorescence with cell nuclei stained for a marker of cell differentiation. In the case studies shown here, we use both myogenin and pericentrin staining. The presence of myogenin indicates that cells have differentiated but not necessarily fused [10,11], while changes in the shape of pericentrin expression within the nucleus indicate incidences of cell fusion [12]. A further measure of myogenic potency is the cell’s ability to form neuromuscular junctions (NMJs) in the presence of neurons [13]. Acetylcholine receptors play a key role in NMJ formation [14], and so our workflow includes quantification of the presence of the acetylcholine receptor marker alpha-bungarotoxin (BTX) within myonuclei as a metric of potential for NMJ formation. Metrics of myotube dimensions, alignment and spatial distribution of myonuclei are used to classify cell morphologies, and the quantification of the proportion of actin striations within myotubes are defined as indicators of cell maturity [15].

Here, we provide a summary of the findings of novel case studies that use this toolkit to highlight a range of applications in identifying myogenic potency. We applied our quantitative software to both primary mouse-derived myotubes [16] and hiPSC-SMC-derived myotubes. In primary mouse cells, our analysis provides novel insights into improvements in cell quality and myoblast dynamics with changes in environmental stimuli acting upon *in vitro* cell cultures. Specifically, we examine the effects of changing the proportion of foetal bovine serum (FBS) in differentiation media, differentiating muscle cells in varying proportions of a bespoke neuron differentiation medium [7] and optogenetic training of muscle cells to replicate the effects of innervation [17]. Quantification of hiPS-SMC determined trends in myonuclei recruitment and spatial distribution, as well as the formation of actin striations as cells mature. Since culturing human muscle cells *in vitro* has proven to be less reliable than primary mouse cells, comparing case studies of mouse and human cell development allows us to determine the traits which make mouse models successful and, thus, provide a benchmark against which interventions in human cells can be targeted.

In summary, we introduce a novel tool for quantifying metrics of myotube quality and myoblast fitness in differentiating skeletal muscle cells and demonstrate its use with case studies on both mouse and human cells of high relevance in the field of *in vitro* skeletal muscle culturing. We believe that a shift towards more quantitative approaches is needed to advance the field.

## 2. Methods

### 2.1. Implementation

All software files used in our workflow, including ImageJ macros for preprocessing and binarizing images, as well as instructions on use, are available at [18]. Documentation is also provided in the electronic supplementary material. Exemplar images and video files are also available at [19] with examples: live image tracking and analysis of mouse and hiPSC-derived cells, static images of cells stained with myogenin and with pericentrin, and a set of static images stained with pericentrin and BTX. Live cell tracking requires bright-field video images in a format supported by MATLAB software (including Tiff, PNG, JPEG, Gif and PNG). Static image analysis requires image file formats supported by the ImageJ Bio-Formats Importer plugin.

### 2.2. *In vitro* primary myotube culture

*In vitro* myotubes were cultured from primary mice myoblasts as described previously [20]. Briefly, hind limb muscles were isolated from 5- to 7-day-old mice pups, minced and digested for 1.5 h at 37°C using 0.5 mg ml^−1^ collagenase (Sigma) and 3.5 mg ml^−1^ dispase (Gibco) in phosphate-buffered saline (PBS). Subsequently, filtered cell suspension was plated in Iscove's modified Dulbecco's medium (IMDM; Invitrogen) for 4 h in the incubator (37°C, 5% CO_2_). To purify cells from fibroblasts and other contaminating cell types, only non-adherent myoblasts were collected and centrifuged. Cells were resuspended in growth medium (IMDM + 20% FBS + 1% chick embryo extract + 1% penicillin–streptomycin) and plated onto 1 : 100 Matrigel (RD) coated ibidi dishes. After 4 days, medium was changed to trigger differentiation. Depending on the experimental condition, standard muscle differentiation medium (IMDM + 1% penicillin–streptomycin) and neuronal medium (N2B27 medium: 50% Dulbecco's Modified Eagle Medium (DMEM)-F12, 50% Neurobasal + 1X N2 + 1X B27 + 50 μM b-Mercaptoethanol + 1% penicillin–streptomycin) were mixed 1 : 0, 1 : 1 or 0 : 1, containing 0, 2, 5 or 10% horse serum. After 1 day of differentiation, a thick layer of 1 : 1 Matrigel was added on the top of the forming myotubes and 100 ng ml^−1^ agrin was added to the culture medium. Cells were cultured up to 7 days at 37°C, 5% CO_2_.

The Rodent Facility of the Instituto de Medicina Molecular João Lobo Antunes maintains high standards of animal welfare and promotes a responsible use of animals, hence supporting the state-of-the-art animal-based research.

The Rodent Facility has a licence of establishment for breeding and animal use, issued by the Portuguese competent authority, Direcção-Geral de Alimentação e Veterinária (DGAV), since 2013. This facility complies with Portuguese law Decreto-Lei 113/2013, transposed from the European Directive 2010/63/EU, and follows the European Commission recommendations (2007/526/EC) on housing and care of animals and the Federation of European Laboratory Animal Science Associations (FELASA) guidelines concerning laboratory animal welfare (Ethics approval number AEC 2014 03 EG).

### 2.3. Optogenetics

The methods for application of optogenetic stimulation to mouse primary muscle cells are described in detail in [17].

#### 2.3.1. *In vitro* human-induced pluripotent stem cell myotube culture

The expansion of hiPSCs (a gift from Rocha S. Lab) was performed in 1 : 50 Matrigel-coated 6-well plate with mTeSR Plus medium (Stem Cell Technologies) + 0.5% PS mTeSR medium. To generate hiPSCs-derived myogenic progenitors, a protocol published by van der Wal *et al.* [1] was used as a starting point. Expandable myogenic progenitors for cryopreservation were produced as described [1] to further differentiate them into multi-nucleated myotubes.

For myotube differentiation, myogenic progenitors were expanded in proliferation medium (DMEM high glucose, 10% FBS, 10 ng ml^−1^ fibroblast growth factor (FGF), 1% penicillin, streptomycin, and glutamine (PSG)) in Matrigel-coated dishes (1 : 50), until they reached 70% confluence. When the cells were confluent, they were either passaged or differentiation was initiated by changing the medium to differentiation medium (DMEM-F12, 2% knockout serum replacement (KOSR), 1× Insulin-Transferrin-Selenium-Ethanolamine (ITS-X), 1% PhoenixSongs Biologicals (PS)) supplemented with differentiation factors (1 μM Chir 99021, 10 μM SB431542, 10 μM Prednisolone). The medium was changed every other day. For live imaging, cells were imaged on day 1 and day 2 of differentiation for live image acquisition. On day 4, a thick layer of 1 : 1 Matrigel was added on top of forming myotubes. For static acquisition, cells were cultured up to 8 days.

### 2.4. Live imaging

Live imaging was performed using an inverted microscope (Zeiss Cell Observer SD or Zeiss Cell Observer) in widefield mode, using a 20× phase air objective (Plan-Apochromat Ph2 NA 0.80 or EC Plan-Neofluar Ph 2 NA 0.50, respectively). Cells were imaged 2 h after adding the respective differentiation medium; 3 × 3 tiles with 10% overlap with 2 × 2 binning were taken every 5 min for a minimum of 12 h. Images were stitched using Zen Blue software and stacked into 30 min videos for further quantitative analysis.

### 2.5. Immunostaining and static imaging

Cells were washed once with PBS and fixed in 4% paraformaldehyde (PFA) for 10 min at room temperature. To stain for acetylcholine receptors, cells were incubated with 1 : 50 Alexa Fluor 488 alpha-bungarotoxin (BTX, Invitrogen, B13422) in PBS for 20 min. Cells were permeabilized (PBS + 0.5% Triton) for 5 min and blocked in blocking buffer (10% in goat serum in PBS + 5% Bovine Serum Albumin (BSA)) for 1 h at room temperature. Primary antibodies (1 : 200; anti-α-actinin (sarcomeric) mouse monoclonal antibody, Sigma Aldrich and A7732) were diluted in blocking buffer containing 0.1% saponin, and cells were incubated at 4°C overnight. Dishes were washed 2× for 5 min in PBS under agitation. Secondary antibodies (1 : 400; goat anti-mouse IgG Alexa Fluor 555, Thermo Fisher Scientific, A21424; goat anti-rabbit IgG Alexa Fluor 647, Thermo Fisher Scientific, A21245) in blocking buffer were incubated in the presence of DAPI (1 : 10 000/1 µg ml^−1^) for 1 h. Dishes were washed twice, and 200 µl Fluoromount-G (Invitrogen) was added on top of cells. Samples were stored at 4°C.

Dishes were imaged with an inverted fluorescent microscope (Zeiss Cell observer) using a 40× phase air objective (EC Plan-NeoFluar NA 0.75). z-stacks of 1 μm were taken as 3 × 3 tiles with 10% overlap and 1 × 1 binning. Subsequently, images were stitched in Zen Blue.

For hiPSC-derived myotubes, cells were washed once with PBS and fixed in 4% PFA for 10 min at room temperature and washed 3 × 5 minutes with PBS. Cells were permeabilized (0.1% Triton X-100 in PBS) for 5 min and blocked in blocking buffer (3% BSA + 0.1% Tween-20 in PBS (PBS-T)) for 1 h at room temperature. Primary antibodies (1 : 100 anti-α-actinin (sarcomeric) mouse monoclonal antibody, Sigma Aldrich and A7732; 1 : 100 anti-myogenin rabbit polyclonal antibody, Abcam, ab219998; 1 : 200 anti-pericentrin rabbit polyclonal antibody, Abcam, ab4448) were diluted in blocking buffer and incubated overnight at 4°C. Dishes were washed 3× for 5 min in PBS-T under agitation. Secondary antibodies (1 : 400 goat anti-mouse IgG Alexa Fluor 488, Thermo Fisher Scientific, A11029; goat anti-rabbit IgG Alexa Fluor 555, Thermo Fisher Scientific, A21429; and 1 : 50 Phalloidin Alexa Fluor 647 Phalloidin, A22287) in blocking buffer were incubated in the presence of DAPI (1 μg ml^−1^) for 1 h at room temperature. Dishes were washed 3× in PBS-T under agitation, and 150 μl Fluoromount-G (Invitrogen) was added on top of cells.

### 2.6. Metrics

#### 2.6.1. Cell behaviours: myoblast motion

Myoblast motion was determined from the positions of cell centres in frames of live, bright-field videos from day 0 to day 1 of the differentiation phase. Skeletal muscle cell tracking involves the fusion of cells into multi-nucleated myotubes; thus, a successful algorithm must accurately distinguish between single-nuclei myoblasts and multi-nucleated myotubes. Once myoblasts and myonuclei were discretized, myoblasts were tracked between frames using MATLAB software.

Each video frame was binarized to determine cell regions, and small regions were removed. Regions longer than a manually chosen threshold length were determined to be myotubes and moved to a separate image. Centroids of myoblast regions were labelled for tracking between frames. An optional manual selection tool was included for the initial frame to add myoblasts missed by the discretization process (such as overlapping myoblasts) or remove myotubes falsely labelled as myoblasts.

A myoblast centroid appearing within a manually set threshold radius of a labelled centroid in the preceding frame is labelled as the new position for the myoblast. If more than one centroid is within this radius, centroids are matched to the nearest myoblast from the previous frame. If no cell centroids appear in the subsequent frame, the position of the cell remains stationary. If no centroid appears for three consecutive frames, the cell is removed.

The software measures nearest neighbour and Voronoi distances between myoblast centroids.

Cell tracking was used to obtain metrics of myoblast direction and motion as follows.

(1) Speed of cell motion

$$S_{n}=\frac{\sum d_{\left(n,t\right)}}{\sum t},$$

where *d_(n,t)_* is the Euclidean distance between centroids of myoblast *n* in consecutive frames and *t* is the length of time between frames in which myoblast *n* is present.

(2) Persistence of cell motion

$$P_{n}=\frac{D_{n}}{\sum d_{\left(n,t\right)}},$$

where *D_n_* is the Euclidean distance between the centroids of myoblast *n* in the first and last frames in which it appears.

(3) Net direction of cell motion

$\Theta_{n}$ represents the angle between the vertical vector and the vector joining the centroids of myoblast *n* in the first and last frames in which it appears.

(4) Rotational velocity

$$V\theta_{n}=\frac{\sum \theta_{\left(n,t\right)}}{\sum t},$$

where $\theta_{\left(n,t\right)}$ is the smallest angle between the vectors joining the centroids of myoblast *n* between two consecutive pairs of frames.

(5) Variation in angular motion

The standard deviation in velocity of angular motion for all tracked myoblasts within an image (σVθ).

(6) Speed of rotation

$$S\theta_{n}=\frac{\sum \left|\theta_{\left(n,t\right)}\right|}{\sum t}.$$

Distinct from rotational velocity, the absolute value of angular rotation between frames is calculated.

(7) Rotational persistence

$$P\theta_{n}=\frac{\left|V\theta_{n}\right|}{S\theta_{n}}.$$

(8) Rate of proliferation

Cell division was visible in bright-field images and, thus, the proliferation rate was measured by manually counting the number of divisions in a 1 mm^2^ field of view over the course of a 2 h video (12 frames per hour).

#### 2.6.2. Cell behaviours: fusion dynamics

A count of unfused myoblast cells and fused myonuclei in stained, static images at timepoints throughout cell differentiation informs of the dynamics of cell fusion over time, allowing comparison of absolute and proportional densities of cell types between models.

All nuclei are stained with a DAPI staining. DAPI stained images are binarized, regions smaller than 1/30th of the mean nucleus area were removed entirely, and regions smaller than half of the mean nucleus area were not included in cell counts. Software was developed to determine the state of differentiation of a given nucleus.

Our previously developed workflow [7] (included as an option in the current workflow) assumed nuclei to be myonuclei if regions in which DAPI-stained nuclei overlap with α-actinin stained myotube regions. Here, we determine the status of cell differentiation based upon staining of myonuclei. Muscle cell nuclei are stained for either myogenin or pericentrin as markers of cell differentiation [12].

The proportion of pixels within each nucleus region containing myonuclei staining is calculated, and nuclei with staining above a threshold proportion are determined to be myonuclei. Smaller nuclei regions (less than or equal to 2 × mean single nucleus pixel area) are designated as single nuclei, while larger regions (greater than 2 × mean single nucleus pixel area) are designated as multiple nuclei (clustering of nuclei is a common phenotype in early-stage myotube differentiation). The number of nuclei in multiple nuclei regions is estimated by dividing the total area by the mean of the single nuclei area.

For binary markers of cell differentiation such as myogenin, the myonuclei count is subtracted from the total nuclei count to provide the number of myoblasts. For markers in which the staining expands continuously, such as pericentrin, an effective threshold for the proportion of staining co-locating with a nucleus is set to maximize the capture of myonuclei. Here, myogenin staining is used for studies using mouse primary cells, while pericentrin is used for hiPSC-SMCs. Nuclei discretization software was validated via comparison with manually labelled nuclei for both myogenin and pericentrin staining.

Nuclei counts are used to determine metrics of myoblast density (nuclei mm^−2^), myonuclei density (nuclei mm^−2^), total nuclei density (nuclei mm^−2^) and FI (myoblast density/total nuclei density). Dead cells present as small regions of high-intensity DAPI fluorescence and were quantified by recording regions with an area smaller than half of the mean nuclei region size in an image field of view.

#### 2.6.3. Quality indicators: cell dimensions and alignment

Cells observed in this study were cultured on two-dimensional surfaces and, thus, determining the area filled by myotubes within an image informs of the total density of myotubes. Dividing the total myotube area by the length of the central skeleton of each myotube provides an estimate of the average width of myotubes within an image.

From the central skeletons of binarized myotubes in an image, we determine the direction of alignment and variation in the direction of alignment of myotubes. The amount of overlap between myotube cells, especially during later stages of differentiation, adds challenge to determining the alignment of whole myotubes. Overlapping myotube skeletons were split into smaller sections by removing the point at which cells cross and linking neighbouring cell sections as those with a similar directional alignment.

#### 2.6.4. Quality indicators: myonuclei spatial distribution

Distance between myonuclei is regulated by a combination of the rate of cell fusion and inter-nuclei forces pushing nuclei apart. A semi-automated approach is applied to determine the mean and standard deviation of distance between nuclei within cells by manually selecting the centre points of myonuclei in a random sample of myotubes within images. Uniformity of the distribution of myonuclei within myotubes is assessed using the coefficient of variation


$$\text{CV}=\frac{\sigma_{dist}}{\mu_{dist}},$$

where $\sigma_{dist}$ is the standard deviation in distance between nuclei within a cell and $\mu_{dist}$ is the mean distance between nuclei within a cell. A lower coefficient of variation tends towards the uniformity of distribution.

#### 2.6.5. Quality indicators: myotube structures

A metric of the proportion of myotubes with actin striations was obtained by manually labelling samples of cells within an image as containing no striations, less than 50% of the cell length containing striations and greater than 50% of the cell length containing striations.

To quantify nuclei acetylcholine receptors, images stained with DAPI and acetylcholine receptor-binding α-BTX are analysed. Myotube nuclei regions are segmented from DAPI stained images using ImageJ software. The mean value of BTX pixel intensity within each myotube nucleus region is calculated in MATLAB. A value for the background BTX fluorescence intensity is determined from the modal bin value of a histogram of nuclei mean BTX intensities. High mean nuclei BTX intensity, indicating the presence of acetylcholine receptors, is defined as being any value over two times this background value. The proportion of nuclei with high mean BTX intensity is then calculated for each image.

#### 2.6.6. Validation of metrics

Metrics calculated from automated segmentation and tracking of cells and cell nuclei were validated by comparison with metrics derived from images with manually defined cell/nuclei positions. For metrics of myoblast motion, the centre positions of all visible myoblast cells within a set of images were marked manually, and the position of each cell was tracked manually over six frames (recording 25 min of cell motion). $S_{n}$, $P_{n}$, ${S\theta }_{n}$*,* $V\theta_{n}\;\;and\;\;P\theta_{n}$ were calculated from the manually labelled position data and compared with values calculated via automatic cell tracking. Cell behaviour validation was conducted using three different culture environments (2% serum, 5% serum and 2% serum with N2B27 neuron differentiation medium). Nuclei segmentation in stained images, used to calculate myonuclei count, myoblast count and FI, was validated by comparing counts of nuclei labelled manually with those labelled by our software. Regions of interest (250 μm^2^) were manually labelled for 30 images with myogenin staining of myonuclei (a total of 573 manually labelled cells) and 30 images with pericentrin staining of myonuclei (a total of 628 manually labelled cells). Images used for validation were taken at day 7 of differentiation in hiPSC-SMCs to allow a relatively high density of nuclei in each image. The median and RMSE of proportional differences between manual and automated nuclei counts for all 30 images were used to assess the effectiveness of automated labelling.

Since myoblast proliferation, distance between nuclei and proportion of striated cells are recorded manually, verification of methods would be superfluous.

### 2.7. Statistical analysis

Statistical significance was assessed by applying the Wilcoxon rank sum test to data using MATLAB software. In figures, * is assigned to *p*-values ≤ 0.05 and ** to *p*-values ≤ 0.01.

## 3. Results

We present case studies of insights into muscle cell differentiation in muscle cells derived from, first, primary mouse cells and, second, from hiPS cells.

### 3.1. Validation

**Figure 1.**
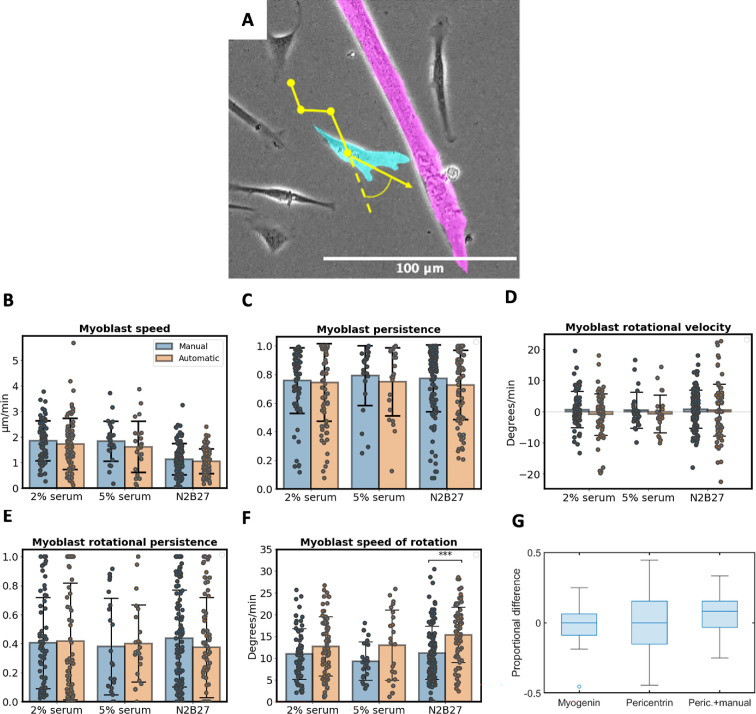
Validation of automated cell metric analysis. (A) Bright-field image of mouse myotube (magenta) and myoblast (cyan) with overlayed illustration (yellow) of myoblast motion over three frames and angle of rotation (coloration applied in post-processing using segmented cell masks). (B–F) Myoblast behaviour metrics for cells in medium with: 2% serum (*n* = 69 cells), 5% serum (*n* = 115 cells). Comparing metrics derived from tracking manually labelled (left-hand columns) and automatically labelled (right-hand columns) myoblast positions over six frames for myoblast speed (B), persistence of motion (C), rotational velocity (D), persistence of rotation (E) and rotational speed (F). Median ± 1 standard deviation shown. (G) Proportional difference between automatically and manually labelled myonuclei counts from 30 images with myonuclei stained with myogenin, pericentrin and pericentrin with an additional manual element.

A comparison of metrics of myoblast motion (figure 1) shows no significant differences between values calculated from manually and automatically labelled cell positions for all measured metrics in all three culture conditions except for cell rotational speed. Cell rotational speed was found to be significantly greater in cells labelled automatically for measurements in N2B27 neuron differentiation medium (*p* = 4 × 10^−6^). This suggests that the absolute value of change in cell direction over time may be too sensitive to measure using our software and, thus, has been omitted from our analysis of cell behaviours below.

The effectiveness of automated labelling of myonuclei in static, stained images was assessed by a comparison with manually labelled nuclei (figure 1F). For myonuclei labelled with myogenin, the median proportional difference between manual and automated labelling was 0% with an RMSE of 13.5%. Myonuclei labelled with pericentrin also produced a median proportional difference between manual and automated of 0%, but an RMSE of 24.6% was deemed too large to provide reliable estimates of myonuclei density. To improve accuracy, a manual element was introduced to select individual myonuclei and myoblast nuclei which have been mislabelled. Adding manual labelling resulted in a slight increase in median proportional difference (8.1%) but reduced RMSE to 14.7%. The largest differences between manually and automatically labelled myonuclei occurred in instances in which aggregations of one nucleus type overlap with another, since the software is designed to assume that large regions of nuclei within an image are of only one type.

### 3.2. Quantitative image analysis reveals differences in primary mouse-derived muscle cell morphology when differentiated with different stimuli

**Figure 2.**
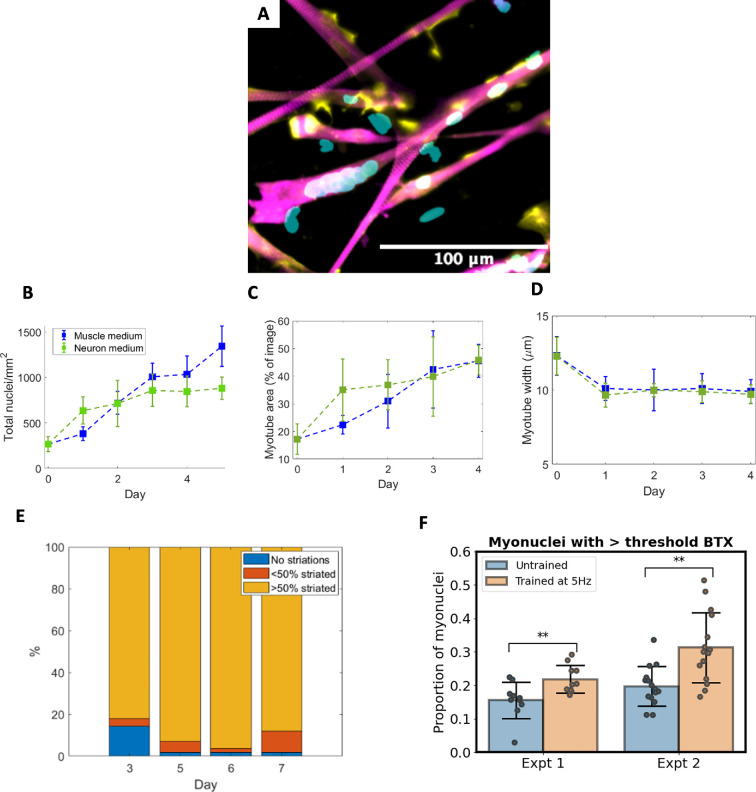
Quantitative analysis of mouse primary cell-derived muscle cell differentiation. (A) Visualization of myotubes stained with α-actinin (magenta), DAPI stained nuclei (cyan) and BTX staining (yellow), an indicator of acetylcholine receptor concentration. (B) Change in myonuclei per mm^2^ over time for cells in standard muscle differentiation medium and N2B27 neuron medium. (C) Proportional area of myotubes per image over time and (D) mean myotube width over time for cells in standard muscle differentiation medium. (E) Proportion of cells exhibiting actin striations from days 3 to 7 of differentiation for cells cultured in standard muscle medium. (F) Proportion of myonuclei with mean BTX staining higher than the threshold for untrained myotubes and myotubes exposed to optogenetic stimulation at 5 Hz for two experiments conducted under identical conditions (*n* = 10 images and 15 images for experiments 1 and 2, respectively).

Quality indicators were extracted from static images with DAPI and myogenin staining for nuclei, α-actinin staining of myotubes and BTX staining for acetylcholine receptors (figure 2A) using a semi-automated nuclei selector. Myogenin is needed for the expression of genes required to initiate normal myocyte fusion [10,11]. It provides a binary indicator of whether cells have differentiated and are able to fuse.

The change in density of myonuclei mm^−2^ indicates the amount of cell fusion which has occurred (figure 2B). Our results show that the differentiation of mouse myoblasts into myonuclei had begun even before the introduction of differentiation medium (267 ± 84 myonuclei mm^−2^ at day 0). After differentiation medium is added, cells cultured in standard medium underwent differentiation at a steady rate between days 0 and 3 (1009 ± 150 myonuclei mm^−2^ [2] by day 3), after which the rate of differentiation slowed. Surprisingly, the initial rate of differentiation was faster for cells cultured in neuron differentiation medium than muscle medium (637 ± 148 and 382 ± 74 myonuclei mm^−2^ at day 1 of differentiation, respectively), though the increase in myonuclei slowed after day 1 in the neuron medium trial, resulting in less myonuclei than the standard medium trials by day 5.

Trends in myotube proportional area for cells cultured in muscle medium (figure 2C) mirror the change in myonuclei density, with a steady increase in myotube formation between days 0 and 3 (mean 25.2% increase), then levelling off from day 3 onwards. The larger initial increase in myotube area seen in cells cultured in neuron medium matches the trend seen in myonuclei density, although by day 4, the myotube areas of cells in both media are similar despite the significantly lower myonuclei density found in cells cultured in neuron medium. No significant differences were found between the mean diameter of myotubes for different culture media (figure 2D). Cell diameter was shown to decrease from approximately 12 to 10 μm during the first day of differentiation and then remained consistent thereafter.

Our previous results [7] have shown that myonuclei in myotubes cultured in neuron differentiation medium and those cultured with 10% FBS were distributed significantly further apart than those in standard differentiation medium at day 5 of differentiation. The coefficient of variation of myonuclei distribution was higher in cells cultured in standard muscle differentiation medium than in neuron differentiation medium, indicating that nuclei are distributed less uniformly in standard medium and there was no significant difference in myonuclei uniformity with changes in FBS concentrations.

Mature myotube cells develop striations, complex structures of actin and myosin, which are integral for muscle function. Quantification of actin striations shows the majority (81%) of myotubes to have striations by day 3 of differentiation (figure 2E) with almost all striated from day 5 onwards.

The pre-patterning of muscle fibres in anticipation of NMJ formation occurs when acetylcholine receptors cluster within the muscle cells [13]. Quantification of the proportion of myonuclei with high densities of acetylcholine receptors may give an indication of how responsive cells will be to the formation of NMJs in each culture condition or when subject to a specific stimulus [17]. Trials in which myotubes modified with optogenetic actuators were trained with a 5 Hz temporal light pattern show a significant (*p* < 0.01) increase in the proportion of myonuclei with BTX staining compared with untrained cells (figure 2F). This indicates increased clustering of acetylcholine receptors in nuclei after training.

### 3.3. Human-induced pluripotent stem cell-derived myoblasts are less persistent and less proliferative than mouse myoblasts

**Figure 3.**
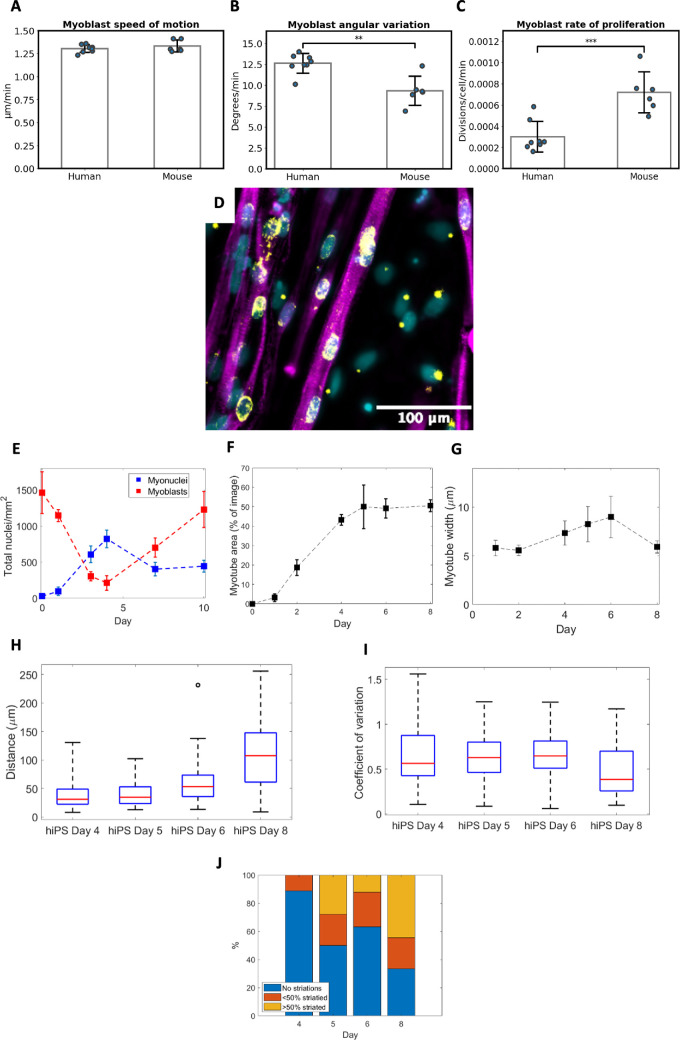
Quantitative analysis of human-induced pluripotent stem cell-derived cells. (A–C) Metrics of myoblast motion for human iPS cells compared with mouse primary cells cultured in differentiation medium with no serum (*n* = 8 and 6 video sets for hiPS and mouse cells, respectively). (D) Visualization of myotubes stained for α-actinin (magenta), DAPI stained nuclei (cyan) and pericentrin staining (yellow) presenting as a single dot in myoblasts and spreading throughout the nucleus in mature myonuclei. (E) Change in myonuclei (blue) and myoblasts (red) per mm^2^ over time. (F) Proportional area of myotubes per image over time. (G) Mean myotube width over time. (H) Mean distance between myonuclei per myotube and (I) uniformity of distribution of myonuclei per cell (measured as coefficient of variation) in which lower values tend to greater uniformity. (J) Proportion of cells exhibiting actin striations from days 4 to 8 of differentiation. For plots E–J, data are from *n* = 10 imaged positions.

Metrics of myoblast motion were compared between hiPSC-SMS-derived myoblasts (*n* = 8 sets of 6 frames with mean of 152 myoblasts tracked per set) and primary mouse myoblasts (*n* = 6 sets of 6 frames with mean of 155 myoblasts tracked per set) cultured in differentiation medium with no added FBS. Myoblast speed (figure 3A) was found to be very similar between cell types, while hiPSC-derived myoblasts displayed significantly higher (*p* < 0.01) angular variation (figure 3B), and therefore lower persistence of motion, and a significantly lower (*p* < 0.001) rate of proliferation (figure 3C) compared with primary mouse-derived cells.

### 3.4. Quantitative image analysis reveals trends in fusion and morphology of human-induced pluripotent stem cell-derived muscle cells during differentiation

The quality indicator metrics of the hiPSC-SMCs described here were extracted from static images with DAPI and pericentrin staining for nuclei, α-actinin staining of myotubes and pericentrin staining of myonuclei using a semi-automated nuclei selector. Pericentrin is a centrosomal protein expressed in myoblasts and myonuclei, presenting in stained images as a dot of fluorescence in myoblasts, which expands with the centrosome as the nuclei differentiate, finishing as a ring of fluorescence surrounding the fully differentiated myonucleus [12].

Analysis of myonuclei densities (figure 3D) shows that, unlike mouse cells, the number of hiPS-derived cells differentiating before the addition of differentiation medium is negligible (28 ± 18 myonuclei mm^−2^ at day 0). Only a small mean increase of 68 myonuclei mm^−2^ occurs during the first day of differentiation, followed by a sharp increase in the rate of differentiation between days 1 and 4 (mean increase of 729 myonuclei mm^−2^). After day 4, the density of myonuclei decreases significantly, indicating cell death. Myonuclei density then remains consistent from day 7 onwards. Myoblast density decreases rapidly from day 0 to day 4 as cells differentiate into myonuclei. After day 4, myoblast density rises steadily. Together, these results indicate that most fusion occurs during days 1−4 of differentiation, and myoblast proliferation continues after this.

Analysis of myotube proportional area (figure 3E) shows that myotube growth follows the same trend as myonuclei density from days 0 to 4. A key difference is that the proportional area of myotubes remains constant from day 5 onwards, despite the loss of myonuclei mentioned above.

Myotube width (figure 3F) was found to be thinner than mouse-derived myotubes (approx. 6 μm on days 1, 2 and 8). The increase in width observed on days 4, 5 and 6 is most probably due to the tendency of myotubes to aggregate together and, thus, two or more myotubes are counted as one here.

In healthy mature muscle cells, myonuclei are distributed uniformly throughout the length of the cell. A uniform distribution of nuclei is hypothesized to be the most efficient for the transport of proteins [21], and so less uniformity would indicate less effective transport. Uniformity of nuclei is disrupted in diseased cells such as Duchenne’s muscular dystrophy [22]. Spatial distribution of myonuclei shows that the distance between myonuclei (figure 3G) remains similar during days 4−5 of differentiation, after which myonuclei move further away from each other (median distances of 31.2 and 107.7 μm at days 4 and 8, respectively). Measurement of the coefficient of variation (figure 3H) shows the uniformity of distribution of nuclei within cells to be consistent between days 4−6 of differentiation (approx. 0.6), after which they move towards uniformity (0.386 at day 8).

Quantification of actin striations in myotubes (figure 3I) reveals that, unlike mouse-derived cells, there is little striation by day 4 of differentiation. Fully striated myotubes develop after this point, but even by day 8 of differentiation, only 55% of myotubes are majority striated.

## 4. Discussion

By applying quantitative analysis to images of *in vitro* muscle cell differentiation, we defined a workflow to extract metrics of myoblast motility, myogenic fusion dynamics, myotube morphology and proportion of actin striations in cells to determine early-stage indicators of cell quality. These metrics were applied to studies of mouse- and human-derived myoblasts to provide insights into cell behaviour and predict cell quality during differentiation.

Currently, the standard metric of success in muscle cell differentiation is the FI, the ratio of myonuclei to total muscle cell nuclei at a given time point. Previous studies suggest that FI can be unrepresentative when offered as the only metric of muscle cell differentiation and, at worst, can misrepresent fusion efficiency [7]. A full list of the metrics provided by our workflow is shown in table 1.

**Table 1.** List of muscle cell metrics applied in workflow.

metric	type	units	symbol
myoblast speed of motion	myoblast behaviour	μm min^−1^	$S_{n}$
myoblast persistence of motion	myoblast behaviour	N/A	$P_{n}$
myoblast net angle of motion	myoblast behaviour	degrees	$\theta_{n}$
myoblast speed of angular motion	myoblast behaviour	degrees min^−1^	${S\theta }_{n}$
myoblast velocity of angular motion	myoblast behaviour	degrees min^−1^	${V\theta }_{n}$
myoblast persistence of angular motion	myoblast behaviour	N/A	${P\theta }_{n}$
variation in myoblast angle of motion	myoblast behaviour	degrees min^−1^	σVθ
rate of myoblast proliferation	myoblast behaviour	divisions cell^−1^ min^−1^	p
myoblasts mm^−2^	fusion dynamics	cells mm^−2^	
myonuclei mm^−2^	fusion dynamics	cells mm^−2^	
total nuclei mm^−2^	fusion dynamics	cells mm^−2^	
fusion Index	fusion dynamics	N/A	FI
dead cells mm^−2^	fusion dynamics	cells mm^−2^	
proportional area of myotubes	myotube dimensions / alignment	N/A	
myotube diameter	myotube dimensions / alignment	μm	
myotube direction of alignment	myotube dimensions / alignment	degrees from vertical	
variation in the direction of alignment	myotube dimensions / alignment	degrees	
mean distance between myonuclei per myotube	myonuclei spatial distribution	μm	D
variation in distance between myonuclei per myotube	myonuclei spatial distribution	μm	$\sigma_{D}$
coefficient of variation of myonuclei distribution	myonuclei spatial distribution	N/A	CV
proportion of myotubes with actin striations		N/A	
proportion of myonuclei with high mean BTX intensity		N/A	

Reduced, or inefficient, myoblast motility will decrease the likelihood of fusion occurring. This has several adverse consequences for cell fusion efficiency and muscle growth [23]. Our workflow includes metrics of myoblast speed, perseverance of motion and rate of proliferation to both characterize cell behaviours and help predict myogenic potency. Quantification of the direction of myoblast motion indicates whether there is directional bias in the myoblast population due to environmental or chemical cues.

Automated counting of myoblast and myotube nuclei allows for the calculation of FI with a manual nucleus labelling tool included to correct any mislabelled nuclei. We also include a count of nuclei significantly smaller than the average area to account for unviable cells (‘dead’ cells). Myonuclei in healthy myotubes have previously been shown [21] to be distributed such that the distance between nuclei is maximized. The reason for this uniformity of distribution is not known, although it is hypothesized that clustering of myonuclei would lead to inefficient maintenance of the myofibre [24]. A breakdown in this uniformity of distribution is a hallmark of many muscle disorders as described by Folker *et al*. [22]. Here, metrics of both the average distance between myonuclei and their uniformity of distribution within myotubes (coefficient of variation) are included.

Automated estimation of myotube diameters from multiple discrete measurements has previously been shown to give inconsistent results [4]. Our workflow calculates global average myotube diameter for an image from total two-dimensional myotube area and total myotube length. While more robust, this method does not provide data on the distribution of cell diameters. Although not required for the case studies described here, our workflow also provides a metric of myotube alignment which may be beneficial when culturing cells with topographical cues [25].

Structural and functional metrics such as the proportion of cells with actin striations and the proportion of cells exhibiting high BTX fluorescence provide comparative quantification of the processes of cell maturity and the potential for NMJ formation [26], respectively.

A key advantage of our workflow is the ability to effectively distinguish between myoblasts, differentiated myonuclei and multi-nucleated myotubes even when cells overlap. In live bright-field images, we achieve this by comparing cell sizes after image segmentation, with cell overlap generally resolving over time. For static images, we record the co-localization of nuclei stainings with specific myogenic markers and label nuclei as belonging to either myoblasts or myotubes. Myogenic markers can be categorized as either markers of differentiation, such as myogenin used here, which present a binary indicator of cell differentiation but do not infer fusion has occurred, or markers of cell–cell fusion, such as pericentrin used here, which present a transformation in distribution as cell fusion is occurring. This progressive change in myogenic marker has the potential to offer further information about the stage of cell maturity. The software also accounts for the overlapping of nuclei regions caused by clustering, an integral part of myotube development.

Quantitative image analysis reveals fundamental differences in the dynamics of *in vitro* differentiation between mouse- and human-derived cells even when differences in timescales for differentiation between species are accounted for. Mouse primary cells begin differentiating instantaneously upon addition of differentiation medium and exhibit a steady increase in cell–cell fusion over subsequent days, while hiPSCs show little recruitment of myonuclei during the first day of exposure to differentiation medium, followed by a rapid increase in differentiation which, in turn, is followed by a significant reduction in myonuclei, presumably from cell death. Myotube formation in hiPSCs was then shown to plateau despite the drop in myonuclei, which would explain the increase in distance between nuclei. A comparison of the proportion of myotubes with actin striations, an indicator of cell maturity, confirms the robustness of the mouse model as most cells are striated by day 3 of differentiation for mouse cells, while striation formation is largely absent until after 4 days in hiPSCs (in concert with the end of myotube formation and the fall in myonuclei). It may be that the ‘slow and steady’ fusion observed in mouse models is more favourable to muscle cell maturation than the ‘boom and bust’ fusion dynamics observed in human *in vitro* cell culture.

A comparison of myoblast motion between human- and mouse-derived cells reveals that, while both cell types move at a similar speed, hiPS cells showed more variation in their angle of motion, implying that they are less efficient in their motion than mouse-derived cells. Mouse-derived cells were also shown to proliferate at a much faster rate per cell. Together, these findings may explain some of the lack of cell differentiation observed in hiPSC-SMCs in the first day of differentiation.

We show specific environmental stimuli of *in vitro* cultures of mouse primary muscle cells which induce significant differences in metrics of myoblast behaviour and myotube quality indicators. Hennig *et al*. [17] previously observed that optogenetic training of muscle cells induces increases in myotube width and a decrease in distance between myonuclei (probably due to increased cell fusion) with no significant difference in the proportion of striated cells. Here, we show that cells which undergo training via optogenetic stimulation have an increase in myonuclei with a high concentration of acetylcholine receptors. The presence of acetylcholine receptor clusters around myonuclei has been shown to be a potential indicator of the ability of a myotube to form NMJs and, thus, achieve innervation [26].

Automated nuclei counting of stained images shows that myonuclei from mouse primary cells are formed steadily when cultured in muscle differentiation medium. This trend is disrupted when culturing in neuron differentiation medium, which exhibits a faster rate of differentiation during the first day but ultimately leads to fewer myonuclei. Myotube formation was shown to initially follow the same trend as myonuclei recruitment for both media, but by day 3 of differentiation, there are similar amounts of myotubes formed in both media. Together, these suggest that culturing muscle cells in neuron differentiation medium does not affect the yield of myotubes but produces cells with significantly fewer myonuclei, an observation supported by the measured increase in distance between myonuclei in neuron medium.

Validation of myoblast behaviours showed that our cell segmentation and tracking software provide similar results to tracking using manual cell positioning for most metrics. The exception being cell rotational speed for two out of the three trials. Rotational speed may be a particularly sensitive metric due to the sum of the absolute value of the difference in angle compounding any differences between automated and manual labelling, which may explain why these significant differences are not seen in rotational velocity, which sums the relative value.

Our study of human-derived cells shows that quantification of both myoblast and myonuclei density over time provides a more robust overview of cell fusion than the FI alone. Total myotube formation can remain constant despite a loss of myonuclei, which suggests that both nuclei densities and the proportion of myotube area are required metrics to gain a complete perspective of trends in cell differentiation.

Our choice of metrics was designed to provide information concerning cell health and efficiency early in differentiation, as well as providing predictions of mature cell quantity and quality as early as possible. Due to our requirement for early indicators, we excluded structural quality indicators such as the peripheral distribution of myonuclei or triad formation, which they develop later in the maturation process.

## 5. Conclusion

Quantitative image analysis of muscle cell differentiation provides a framework for gaining unique insights into the health and quality of muscle cells during the early stages of differentiation. By integrating metrics of myoblast motility, myogenic potency, myotube morphology, myonuclei distribution and myotube striations, our workflow offers a comprehensive and robust approach to assess cell quality at early stages. These metrics not only enable the identification of trends in cell morphology throughout differentiation but also facilitate an understanding of the underlying biological mechanisms. Furthermore, this approach allows for the quantitative comparison of the effects of various cell culture environments, enhancing the ability to predict mature muscle cell quality from an early stage.

## Data Availability

All ImageJ preprocessing macros and code used for cell quantification can be accessed via the following Zenodo repository:[27] (alternatively:[28]) Image data for validation can be found at the following Zenodo repository: [29] Supplementary material is available online [30].
